# Differences in Frontal Network Anatomy Across Primate Species

**DOI:** 10.1523/JNEUROSCI.1650-18.2019

**Published:** 2020-03-04

**Authors:** Rachel L. C. Barrett, Matthew Dawson, Tim B. Dyrby, Kristine Krug, Maurice Ptito, Helen D'Arceuil, Paula L. Croxson, Philippa J. Johnson, Henrietta Howells, Stephanie J. Forkel, Flavio Dell'Acqua, Marco Catani

**Affiliations:** ^1^NatBrainLab, King's College London, London SE5 8AF, United Kingdom,; ^2^Department of Neuroimaging, Institute of Psychiatry, Psychology and Neuroscience,; ^3^Sackler Institute for Translational Neurodevelopment, Department of Forensic and Neurodevelopmental Science, Institute of Psychiatry, Psychology and Neuroscience, King's College London, London SE5 8AF, United Kingdom,; ^4^Danish Research Centre for Magnetic Resonance, Centre for Functional and Diagnostic Imaging and Research, Copenhagen University Hospital Hvidovre, Hvidovre DK-2650, Denmark,; ^5^Department of Applied Mathematics and Computer Science, Technical University of Denmark, Kongens Lyngby DK-2800, Denmark,; ^6^Department of Physiology, Anatomy and Genetics, University of Oxford, Oxford OX1 3PT, United Kingdom,; ^7^Institute of Biology, Otto-von-Guericke-Universität Magdeburg, Magdeburg 39120, Germany,; ^8^Leibniz-Institute for Neurobiology, Magdeburg 39118, Germany,; ^9^Laboratory of Neuropsychiatry, Psychiatric Centre Copenhagen, Copenhagen DK-2200, Denmark,; ^10^École d'Optométrie, Université de Montréal, Montréal, Québec H3T 1P1, Canada,; ^11^Athinoula A. Martinos Center for Biomedical Imaging, Massachusetts General Hospital, Charlestown, Massachusetts 02129,; ^12^Department of Neuroscience and Friedman Brain Institute, Icahn School of Medicine at Mount Sinai, New York, New York 10029,; ^13^College of Veterinary Medicine, Cornell University, Ithaca, New York 14853, and; ^14^Laboratory of Motor Control, Department of Medical Biotechnologies and Translational Medicine, Università degli Studi di Milano, Humanitas Research Hospital, Istituto di Ricovero e Cura a Carattere Scientifico, Milan, 20129, Italy

**Keywords:** comparative anatomy, connectivity, diffusion MRI, evolution, frontal lobe, tractography

## Abstract

The frontal lobe is central to distinctive aspects of human cognition and behavior. Some comparative studies link this to a larger frontal cortex and even larger frontal white matter in humans compared with other primates, yet others dispute these findings. The discrepancies between studies could be explained by limitations of the methods used to quantify volume differences across species, especially when applied to white matter connections. In this study, we used a novel tractography approach to demonstrate that frontal lobe networks, extending within and beyond the frontal lobes, occupy 66% of total brain white matter in humans and 48% in three monkey species: vervets (Chlorocebus aethiops), rhesus macaque (Macaca mulatta) and cynomolgus macaque (Macaca fascicularis), all male. The simian–human differences in proportional frontal tract volume were significant for projection, commissural, and both intralobar and interlobar association tracts. Among the long association tracts, the greatest difference was found for tracts involved in motor planning, auditory memory, top-down control of sensory information, and visuospatial attention, with no significant differences in frontal limbic tracts important for emotional processing and social behaviour. In addition, we found that a nonfrontal tract, the anterior commissure, had a smaller volume fraction in humans, suggesting that the disproportionally large volume of human frontal lobe connections is accompanied by a reduction in the proportion of some nonfrontal connections. These findings support a hypothesis of an overall rearrangement of brain connections during human evolution.

**SIGNIFICANCE STATEMENT** Tractography is a unique tool to map white matter connections in the brains of different species, including humans. This study shows that humans have a greater proportion of frontal lobe connections compared with monkeys, when normalized by total brain white matter volume. In particular, tracts associated with language and higher cognitive functions are disproportionally larger in humans compared with monkeys, whereas other tracts associated with emotional processing are either the same or disproportionally smaller. This supports the hypothesis that the emergence of higher cognitive functions in humans is associated with increased extended frontal connectivity, allowing human brains more efficient cross talk between frontal and other high-order associative areas of the temporal, parietal, and occipital lobes.

## Abstract (precis)

The frontal lobe is central to distinctive aspects of human cognition and behavior. Some comparative studies link this to a larger frontal cortex and even larger frontal white matter in humans compared with other primates, yet others dispute these findings. The discrepancies between studies could be explained by limitations of the methods used to quantify volume differences across species, especially when applied to white matter connections.

## Introduction

The frontal lobe is considered to play an important role in high-level cognitive functions with differences across species (Passingham and Wise, 2012) and is relatively large in humans compared with other vertebrates (Fuster, 1988). When humans are compared with higher primates, however, the results are mixed, with some reporting no difference in the proportion of frontal (Semendeferi et al., 2002) or prefrontal (Schoenemann et al., 2005) cortical volume. This turned more attention to white matter, in line with Zhang and Sejnowski (2000), who proposed that longer white matter fibers are required by larger brains to guarantee efficient communication between distant cortical areas. Smaers et al. (2011, 2017) and Donahue et al. (2018) reported that the prefrontal cortex and white matter were disproportionally greater in humans than higher primates, yet others dispute these findings (Barton and Venditti, 2013; Gabi et al., 2016). This discrepancy in results could be explained by the lack of consensus on anatomical boundary delineation and the limitations of methods adopted (Sherwood and Smaers, 2013). Nonetheless, there appears to be agreement in the literature that an expansion of distributed white matter networks, rather than cortical volume of the frontal lobe, may have had an important role in the evolution of human higher cognitive functions.

In this study, we performed a comparative analysis of the white matter tracts of the frontal lobe using a novel approach based on diffusion tractography. Compared with structural magnetic resonance imaging (MRI) or tissue-sectioning methods that have previously been adopted to study the frontal lobe, tractography offers two main advantages. First, tract volume can be approximated by calculating the space occupied by streamlines that follow the entire trajectory of white matter pathways. When applied to the frontal lobes, this allows us to analyze the large portion of frontal connections extending beyond the anatomical boundaries of the frontal lobe, which has not been taken into account with previous MRI approaches. Second, distinct tract groups and individual pathways can be virtually dissected and analyzed separately (Catani et al., 2002; Thiebaut de Schotten et al., 2012). Frontal lobe connections can be classified into three main tract groups that include projection fibers (linking the cortex with subcortical nuclei and the brainstem), commissural fibers (linking cortical areas between hemispheres), and association fibers (linking cortical areas within a single hemisphere). Association fibres can be further subdivided into intralobar (within the frontal lobe) and interlobar (between frontal and nonfrontal regions) connections (Catani et al., 2012b). Considering that various tracts and groups of tracts play distinct roles in cognition and behavior, a differentiated tract analysis between species may reveal differences in networks underlying uniquely human abilities (Passingham and Wise, 2012).

Diffusion imaging tractography was acquired from 20 human participants *in vivo*, nine nonhuman primates *ex vivo* (five macaques, four vervets) and six macaques *in vivo*. Diffusion data were analyzed using spherical deconvolution, an advanced diffusion modeling technique, which we have previously applied to reconstruct crossing fibers and visualize tracts that are not visible with tensor-based approaches (Dell'Acqua et al., 2010; Thiebaut de Schotten et al., 2011; Catani et al., 2012a; Dell'Acqua and Tournier, 2019). Deterministic tractography was used to calculate the total volume of frontal lobe white matter; frontal association, commissural and projection tract groups, and finally, individual tracts of the association group. Additionally, a nonfrontal tract, the anterior commissure, was included in the analysis to verify that there may exist tracts in the brain that are disproportionally smaller in humans than monkeys. For each brain, frontal tract volume measurements were divided by total hemispheric tract volume to obtain normalized values. MRI voxel-based measurements of frontal cortical and white matter volume were also obtained for comparison with previous studies.

## Materials and Methods

### 

#### 

##### Participants.

Diffusion MRI data (Table 1) were analyzed from 20 human *Homo sapiens* participants *in vivo* (all male; mean ± SD age, 27.9 ± 5.0 years) and three monkey species *ex vivo*: four vervets (*Chlorocebus aethiops*, all male; mean age, 4.1 ± 1.9 years), three rhesus macaques (RMs; *Macaca mulatta*, all male; mean age, 11.2 ± 2.0 years), and two cynomolgus macaques (CMs; *Macaca fascicularis*, all male; mean age estimated as ≥11 years). In addition, six rhesus macaque (all male; mean age, 5.5 ± 0.4 years) datasets were acquired *in vivo* for a comparison between *in vivo* and *ex vivo* tractography results. The human data were acquired with informed consent under the Biomedical Research Centre Atlas Project, approved by the Joint Medical Ethical Committee of the Institute of Psychiatry, Psychology and Neuroscience, King's College London.

**Table 1.** Diffusion MRI acquisition parameters

Group	Resolution (mm^3^)	*b* value (s/mm^2^)	b0 volumes	DWI volumes
Human	2.40 × 2.40 × 2.40	3000	7	60
Vervet 1	0.50 × 0.50 × 0.50	7660	18	256
Vervets 2–4	0.50 × 0.50 × 0.50	3151	16	87
RM	0.50 × 0.50 × 0.50	4310	3	61
CM	0.43 × 0.43 × 0.43	8000	12	119
RM *in vivo*	1.00 × 1.00 × 1.00	1500	10	80

Unless indicated, the monkey datasets were acquired *ex vivo*. CM, cynomolgus macaque; RM, rhesus macaque; DWI, diffusion weighted image.

The four vervet monkeys were obtained from the Behavioral Science Foundation St Kitts and were socially housed in enriched environments. The experimental protocol was reviewed and approved by the Institutional Review Board of the Behavioral Science Foundation acting under the auspices of the Canadian Council on Animal Care. The post mortem data from three rhesus macaque brains were obtained from a research program at the University of Oxford. All procedures and care were performed in accordance with UK Home Office regulations and European Union (EU) guidelines (EU directive 86/609/EEC; EU Directive 2010/63/EU). For details of tissue fixation, see Dyrby et al. (2011) and Large et al. (2016). The two cynomolgus macaque datasets were obtained from the Martinos Center for Biomedical Imaging Boston. All housing, transport, and experimental procedures were approved by the appropriate institutional animal care panels, described by de Crespigny et al. (2005), and the tissue was prepared as described by D'Arceuil et al. (2007).

The macaque *in vivo* datasets were obtained from the Icahn School of Medicine at Mount Sinai (ISMMS) New York. The experimental procedures required for collecting these data were approved by the ISMMS Institutional Animal Care and Use Committee and conformed to the U.S. Public Health Service Policy on Humane Care and Use of Laboratory Animals, the National Institutes of Health Guide for the Care and Use of Laboratory Animals, and Association for Assessment and Accreditation of Laboratory Animal Care accreditation. They were socially housed as a group in an enriched environment. Scanning was performed under light isoflurane anesthesia as described previously by Mars et al. (2011). Anesthesia was induced using ketamine (10 mg/kg, i.m.) and maintained with isoflurane at a low concentration (0.9–1.7% expired; mean, 1.38%). Anesthesia was supplemented with meloxicam (0.2 mg/kg, i.v.) and ranitidine (0.05 mg/kg, i.v.). Monkeys were intubated and ventilated throughout each experiment. Physiological parameters including capnography, inspired and expired isoflurane concentration, SP0_2_, core temperature, heart rate, and blood pressure were monitored and kept constant to maintain normal physiological function.

##### Diffusion MRI acquisition (Table 1).

The human data were acquired on a 3 T Signa HDx TwinSpeed MRI scanner (GE Healthcare) using an echo planar imaging pulse sequence as described by Dell'Acqua et al. (2013). The vervet and rhesus macaque datasets were acquired with a 4.7 T Varian Inova (Varian) scanner using the protocol described by Dyrby et al. (2011); the cynomolgus macaque data were acquired with a 4.7 T Oxford magnet interfaced to a BioSpec Avance console (Bruker) according to the parameters indicated by D'Arceuil et al. (2007). The *in vivo* rhesus macaque datasets were acquired with a Skyra 3 T scanner (Siemens) with a custom-built 8-channel phased-array coil, with a single-loop local transmit coil (Windmiller Kolster Scientific). Spin echo pulse sequences were used to acquire the *ex vivo* monkey datasets, whereas the *in vivo* monkey datasets were acquired using an echo planar imaging sequence. The diffusion MRI acquisition parameters for all species are summarized in Table 1. The anatomical accuracy and reproducibility of postmortem diffusion MRI has previously been validated using axonal tracing (Dyrby et al., 2007; Jbabdi et al., 2013; Cerliani et al., 2017; Donahue et al., 2016).

##### Diffusion MRI and tractography processing.

All steps from preprocessing to tractography tract dissections were performed in the native space of each individual brain. Data were inspected for artifacts visually and with the ExploreDTI outlier profile tool. Data from one diffusion direction in cynomolgus macaque 2203 were removed due to severe artifacts. The human diffusion data were corrected for head motion and eddy current distortions and registered to a non-diffusion-weighted reference image using ExploreDTI (www.exploredti.com). The *ex vivo* data did not undergo these corrections, as they were scanned using a spin echo sequence that is robust to eddy current and geometric distortions. For the *in vivo* macaque data, eight averages per brain were acquired, four with left–right phase-encoding direction and three with right–left, to facilitate correction for distortions along the phase-encoding direction. After correction for susceptibility-induced off-resonance field effects using the tool Topup (Andersson et al., 2003) as implemented in FSL, datasets were registered and corrected for motion and eddy currents with the FSL Eddy tool (Andersson and Sotiropoulos, 2016).

For all datasets, the fiber orientation distribution function was estimated with StarTrack (www.natbrainlab.co.uk) using the damped Richardson-Lucy algorithm for spherical deconvolution as described by Dell'Acqua et al. (2010). Deterministic tractography was performed in each brain using the Euler algorithm in StarTrack (Dell'Acqua et al., 2013). A whole-brain approach was used, with one seed point per voxel and one streamline generated for each peak of the fiber orientation distribution function above the set anisotropy threshold. Because the *ex vivo* data had varying levels of noise and voxel sizes, spherical deconvolution and tractography parameters were determined experimentally for each group to maximize the ability to resolve crossing fibers and minimize spurious fiber directions (Table 2). Anisotropic power maps (Dell'Acqua et al., 2014) were generated for anatomical reference using StarTrack. The dissections were performed by R.L.C.B., M.D., and P.J. under the supervision of an expert anatomist (M.C.).

**Table 2.** Spherical deconvolution and tractography parameters

Group	α	No. iterations	Angle (°)	Absolute	Relative (%)	Length (mm)
Human	0.25	1000	30	0.40	4	20–400
Vervet 1	0.50	1000	45	0.20	5	10–400
Vervets 2–4	0.10	2000	45	0.20	5	10–400
RM	0.10	3000	45	0.20	5	10–400
CM 2104	0.15	2000	35	0.15	5	10–400
CM 2203	0.38	2000	40	0.18	5	10–400
RM *in vivo*	1.00	1500	35	0.15	5	10–400

Unless indicated, the monkey datasets were acquired *ex vivo*. The above parameters are explained fully by Dell'Acqua et al. (2013). α, Shape factor of the fiber response function; No. iterations of the spherical deconvolution algorithm; Angle, maximum angle threshold between adjacent voxels; Absolute, a tractography stopping threshold based on the absolute value of the hindrance-modulated orientational anisotropy index; Relative, a stopping threshold for tractography set to a percentage of the maximum lobe amplitude of the fiber orientation distribution function; Length, is the length threshold for streamlines. CM, cynomolgus macaque; RM, rhesus macaque.

##### Tractography analysis (Fig. 1 and 2).

The frontal white matter as a whole was dissected in TrackVis (www.trackvis.org) using an inclusion region of interest of the frontal lobe, as defined in humans by the standard MNI152 nonlinear sixth generation MRI atlas segmentation (Collins et al., 1999) and in vervets and macaques by the INIA19 MRI atlas (Rohlfing et al., 2012). These cortical atlas regions were coregistered to anisotropic power maps in the native space of each brain using Advanced Normalization Tools (ANTs; www.picsl.upenn.edu/software/ants). This was done separately for each hemisphere. To isolate the frontal association pathways, exclusion regions were drawn manually to remove any streamlines traveling to the opposite hemisphere (i.e., commissural connections), subcortical nuclei, cerebellum, or brainstem (i.e., projections). Intrafrontal streamlines were defined similarly but with the additional condition that both ends of the streamlines be within the frontal lobe region of interest. The frontal projection pathways were defined for each hemisphere using one region encompassing the basal ganglia, thalamus, and internal capsule, and a second region of the frontal cortex. Frontal commissural pathways were defined to include all streamlines connecting the left and right frontal cortices, and any streamlines not belonging to the corpus callosum were manually removed. The cerebellar white matter and the volume of projection fibers below the level of the pons were excluded from the final volume analysis.

**Figure 1.**
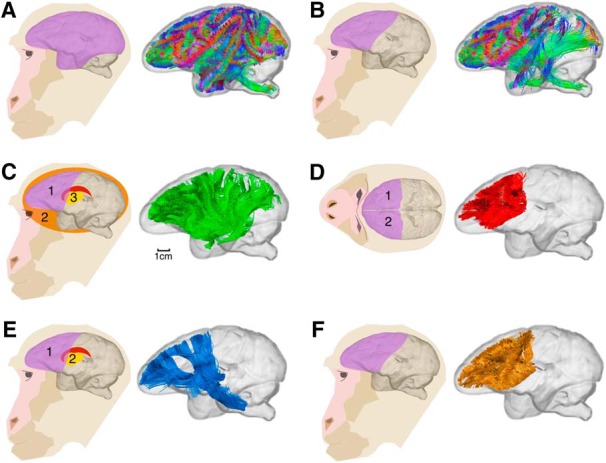
Pipeline for dissection of the association, commissural, projection, and intrafrontal tracts, illustrated in a single macaque brain. ***A***, An inclusion region of the whole left or right hemisphere was used to extract all hemispheric connections. Exclusion regions (not pictured) were used to remove artifactual streamlines coursing through the contralateral internal, external, and extreme capsules. ***B***, From the set of streamlines in each hemisphere defined in ***A***, an inclusion region of the frontal lobe was used to select only streamlines passing through the frontal lobe, including those extending between frontal and nonfrontal regions. ***C***–***F***, These frontal lobe connections were then further separated into the following groups: association fibers, using an inclusion region of the frontal lobe (1) and exclusion regions in the midsagittal section (2) and subcortical nuclei (3); ***C***); commissural fibers, using the two frontal lobes (1, 2) as inclusion regions (***D***); projection fibers, using one inclusion region of the frontal lobe (1) and one in the brainstem, thalamus and internal capsule (2; ***E***); and intrafrontal association fibers (***F***). Intrafrontal fibers were defined with the condition that both ends of the streamline must be within the frontal lobe region of interest. The same approach was used in all species.

**Figure 2.**
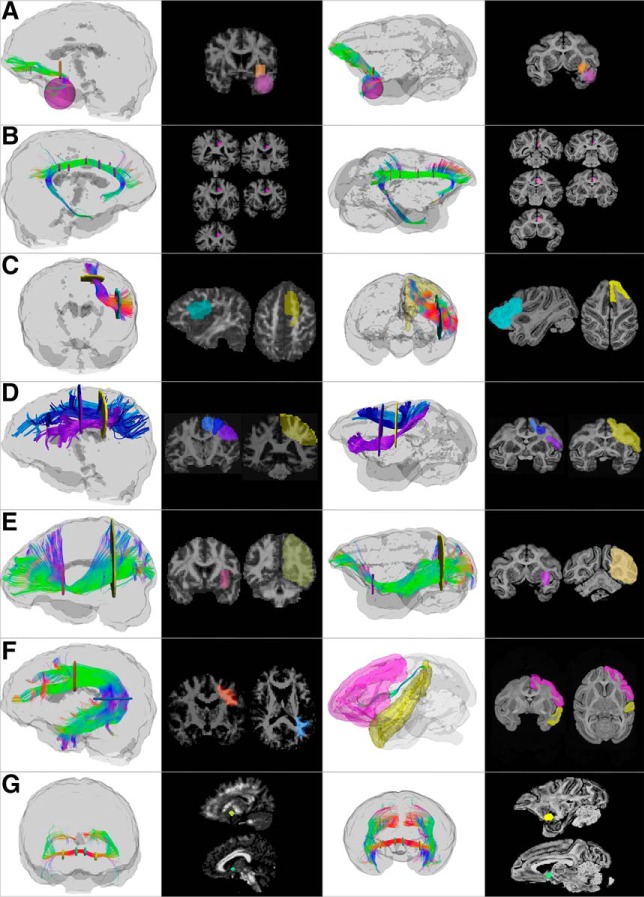
Regions of interest used to dissect individual tracts in the human (two left columns) and monkey (two right columns) brain. For each example, 3D reconstructions and 2D sections are shown. In addition to the regions depicted here, exclusion regions were used in the midsagittal plane, brainstem, subcortical nuclei, and internal capsule to exclude commissural and projection tracts and remove individual spurious streamlines. ***A***, Uncinate fasciculus (lateral view). Inclusion regions of interest are placed in the anterior temporal lobe (pink) and external/extreme capsules (orange). ***B***, Cingulum (medial view). A single inclusion region (pink) on multiple coronal slices along the cingulate gyrus is used to ensure that all the short projections of the dorsal cingulum are included. ***C***, Frontal aslant tract (anterior view). An inclusion region (light blue) is placed in the white matter medial to the inferior frontal gyrus in the sagittal plane. In humans, a second inclusion region (yellow) is placed in the white matter inferior to the superior frontal gyrus in the axial plane, whereas in monkeys, an atlas-defined region of the superior frontal gyrus is used as the second region to include all streamlines projecting to the medial frontal regions. Exclusion regions were then placed in the frontal pole. ***D***, SLF (lateral view). Posteriorly, one inclusion region (yellow) is placed in the parietal lobe in line with the superior aspect of the central sulcus, whereas anteriorly three separate inclusion regions are used for each of the three branches: SLF I (light blue), II (dark blue), and III (purple), all in a coronal plane passing through the precentral gyrus. Exclusion regions are used in the temporal and occipital lobe in both humans and monkeys. ***E***, Inferior fronto-occipital fasciculus (lateral view). One inclusion region is used in the external/extreme capsules (pink) and one in the anterior border of the occipital lobe (yellow); both are in the coronal plane. ***F***, Arcuate fasciculus, long segment. In the human, one inclusion region (orange) is placed in the coronal plane just anterior to the central sulcus, and one inclusion region in the axial plane inferior to the temporoparietal junction (blue). In the monkey, to be as inclusive as possible, atlas-defined regions of the frontal lobe (pink mask) and superior temporal gyrus (yellow mask) were also used as inclusion regions of interest. In addition to the inclusion regions pictured here, exclusion regions were placed in the external/extreme capsules and the white matter of the superior temporal gyrus to remove the middle longitudinal fasciculus, and in the white matter medial to the supramarginal gyrus to remove SLF fibers. ***G***, Anterior commissure. Two inclusion regions were used to capture the compact bundle of the anterior commissure as it crosses the midline. Each region has two slices in the sagittal plane on either side of the midline, one more medial (green), one placed more laterally (yellow). Exclusion regions were used to remove spurious streamlines forming part of the fornix, anterior thalamic projections, and other projections from the brainstem.

Manual dissections of individual frontal association tracts were performed. The tracts included in our analysis were the cingulum, uncinate fasciculus (UF), frontal aslant tract (FAT), three branches of the superior longitudinal fasciculus (SLF), inferior fronto-occipital fasciculus (IFOF), and the long segment of the arcuate fasciculus (AF). In addition, the anterior commissure was dissected as a nonfrontal control tract. Tracts were dissected using manually drawn inclusion and exclusion regions of interest, as illustrated in Figure 2. Where multiple inclusion regions are needed to define a tract, a logical “AND” condition was used, so that only streamlines passing through both regions were included in the result. The atlas by Catani and Thiebaut de Schotten (2012) was used as an anatomical reference for human tracts, and the Schmahmann and Pandya (2006) axonal tracing atlas was used for the macaque and vervet datasets. For all dissections, large regions of interest extending into the white matter were used to ensure all relevant streamlines were captured and to avoid region-placement bias. The regions were then edited if necessary to remove irrelevant streamlines, such as those identified as belonging to another tract or with anatomically implausible trajectories, such as looping. In tracts which are less well described, or less similar in the nonhuman species compared with humans, such as the frontal aslant tract and the arcuate fasciculus, atlas-defined rather than hand-drawn inclusion regions were used first to identify all streamlines projecting to the appropriate regions. The dissections were then refined using regions of interest in the white matter to capture only the streamlines from the given tract. Tractography volume measurements were obtained by calculating the total volume of voxels containing streamlines from the given tract. Normalized volumes were obtained by dividing the tract volume by the total volume occupied by hemispheric white matter streamlines, defined using a region of interest of the whole hemisphere, as shown in Figure 1.

##### Voxel-based volume analysis.

Gray matter (excluding subcortical nuclei) and white matter (excluding cerebellar and white matter below the pons) tissue probability maps from the MNI (Fonov et al., 2009, 2011) and INIA19 (Rohlfing et al., 2012) templates were coregistered to anisotropic power maps in the native space of each brain using ANTS (Avants et al., 2011). A minimum probability threshold of 0.1 was applied and a weighted volume (i.e., volume × tissue probability value) was calculated to obtain measures of gray and white matter volume that are robust to small errors in registration. The frontal volumes were calculated similarly by first applying a frontal lobe mask to the tissue probability maps. To obtain normalized volume measures in each brain, frontal volume fractions were calculated as follows: the frontal cortex volume was divided by the total cortical volume, and the frontal white matter volume was divided by the total white matter volume. Absolute volumes were measured in milliliters, and volume fractions were calculated as percentages.

##### Experimental design and statistical analysis.

For statistical analysis, the data were divided into three groups: humans (*in vivo*, *n* = 20), vervets (*ex vivo*, *n* = 4), and macaques (*ex vivo*, *n* = 5). The sample sizes in this study were determined by the availability of high-quality *ex vivo* data in monkey species. Our statistical analysis was performed on normalized volume measurements averaged across the two hemispheres in each brain individually. To identify whether there were species group differences within the different volume measures (voxel-based frontal white and cortical gray matter, tractography-based frontal white matter, frontal association, projection, commissural, and intrafrontal tract groups, and individual tracts), a one-way Welch ANOVA (Welch, 1951) using an asymptotically distributed *F* statistic was applied with SPSS version 20 (IBM). In the measures with significant species group differences (*p* < 0.05), a Games-Howell *post hoc* analysis was applied to determine the specific differences between species groups (Games and Howell, 1976). Additionally, we compared the group of *in vivo* macaques (*n* = 6) with the *ex vivo* macaque and *in vivo* human data using Welch's *F* followed by Games-Howell *post hoc* tests, as above. The statistical tests used in this study were chosen for being robust to small group sizes and inhomogeneity of variance between groups (Games and Howell, 1976; Clinch and Keselman, 1982). Type I errors are controlled for by the Games-Howell *post hoc* analysis when carrying out multiple comparisons (Games and Howell, 1976). Results are reported as species group mean ± SD. The data presented in this article and the protocols and code used in the analysis will be available to readers upon request to the corresponding author.

## Results

**Figure 3.**
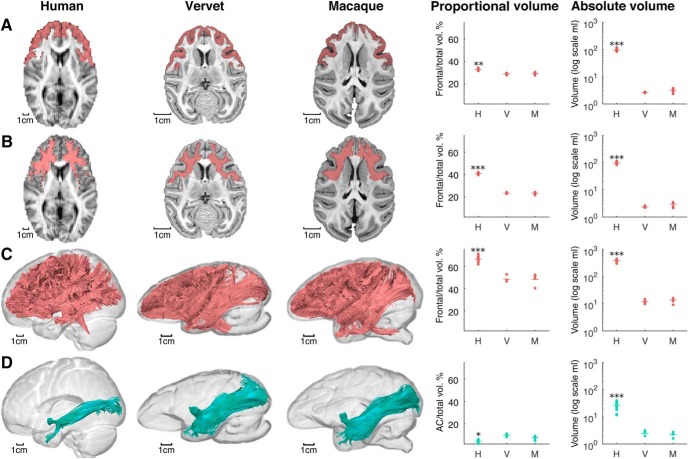
MRI methods for comparing cortical and white matter volumes across species. Images show the rescaled anatomy of representative cases, and graphs display proportional and absolute volumes. Data points represent individual cases, dashes represent species means. H, Humans (*n* = 20); V, vervets (*n* = 4); M, macaques (*n* = 5). ***A***, Voxel-based measures of frontal cortex volume. ***B***, Voxel-based measures of frontal white matter volume. ***C***, Tractography-based measures of frontal tracts volume. ***D***, Tractography-based measures of anterior commissure (AC) volume. *p < 0.05, **p < 0.01, and ***p < 0.001 when comparing humans with either vervets or macaques. For full statistical results, see Results and Table 3.

Figure 3 and Table 3 show the results for proportional and absolute volumes obtained with voxel-based and tractography-based MRI measurements of frontal cortical and white matter. The ANOVA of volume proportions indicated statistically significant differences among the three species groups for the frontal cortex (Welch's *F*_(2,5.88)_ = 46.47, *p* < 0.001), the voxel-based frontal white matter (Welch's *F*_(2,5.65)_ = 1415.65, *p* < 0.001), and the tractography-based frontal white matter (Welch's *F*_(2,5.60)_ = 84.03, *p* < 0.001). Games-Howell *post hoc* analysis showed that human brains had a higher frontal cortex volume fraction (32.69 ± 0.79%) compared with both vervets (28.89 ± 0.79%; *p* = 0.002) and macaques (29.12 ± 1.22%; *p* = 0.004). The differences for the voxel-based frontal white matter volume fraction were even greater between humans (40.80 ± 0.62%) and both vervets (23.33 ± 0.72%; *p* < 0.001) and macaques (23.19 ± 1.04%; *p* < 0.001). Finally, our novel method using tractography to analyze the volume of frontal lobe networks extending throughout the brain also showed a higher volume fraction in humans (66.18 ± 2.56%) compared with vervets (48.16 ± 2.94%; *p* = 0.001) and macaques (47.98 ± 4.54%; *p* = 0.001). No statistically significant differences existed between monkey species in these three measures (Table 3). These results confirm previous voxel-based findings (Schoenemann et al., 2005; Smaers et al., 2010) and indicate that our tractography measures are able to detect simian–human differences in tract volumes. Differences between species were also statistically significant for the absolute measurements of frontal gray matter volume (*F*_(2,9.713)_ = 1122.75, *p* < 0.001), voxel-based frontal white matter volume (*F*_(2,10.48)_ = 1329.29, *p* < 0.001), and tractography-based frontal white matter volume (*F*_(2,13.53)_ = 632.49, *p* < 0.001; Table 3).

**Table 3.** Proportional and absolute frontal volume measurements between species

Volume measures	Human (mean ± SD)	Vervet (mean ± SD)	Macaque (mean ± SD)	*Post hoc* comparisons (*p* values)		
				Human versus vervet	Human versus macaque	Vervet versus macaque
Frontal Cortex (voxel-based)						
Proportion (%)	32.69 ± 0.79	28.89 ± 0.79	29.12 ± 1.22	0.002	0.004	0.938
Absolute (ml)	95.27 ± 8.45	2.68 ± 0.13	3.16 ± 0.57	<0.001	<0.001	0.271
Frontal white matter (voxel-based)						
Proportion	40.80 ± 0.62	23.33 ± 0.72	23.19 ± 1.04	<0.001	<0.001	0.974
Absolute	96.69 ± 7.93	2.33 ± 0.19	2.92 ± 0.59	<0.001	<0.001	0.182
Frontal tracts (tractography)						
Proportion	66.18 ± 2.56	48.16 ± 2.94	47.98 ± 4.54	0.001	0.001	0.997
Absolute	382.60 ± 45.30	11.91 ± 2.04	13.48 ± 2.80	<0.001	<0.001	0.618
Anterior commissure (tractography)						
Proportion	4.59 ± 1.15	9.90 ± 1.30	7.86 ± 1.80	0.004	0.028	0.091
Absolute	26.73 ± 7.91	2.46 ± 0.56	2.18 ± 0.56	<0.001	<0.001	0.754

Frontal and nonfrontal (anterior commissure) volume measures in humans (*n* = 20), vervets (*n* = 4), and macaques (*n* = 5). Descriptive statistics and Games-Howell *post hoc* comparisons between species are given for proportional (normalized by total volume for each measure) and absolute volumes. See Results for Welch's ANOVA statistics.

To examine the implication of humans having proportionally more frontal white matter than monkeys, we analyzed a nonfrontal tract for comparison, the anterior commissure (Fig. 3*D*; Table 3). The ANOVA of the volume fraction of the anterior commissure also indicated statistically significant differences among the groups (Welch's *F*_(2,5.68)_ = 29.95, *p* = 0.001), but in this case, humans had a smaller volume fraction (4.59 ± 1.15%) compared with both vervets (9.90 ± 1.30%; *p* = 0.004 *post hoc*) and macaques (7.86 ± 1.80%; *p* = 0.028 *post hoc*). There was no statistically significant difference in volume fraction of the anterior commissure between the two monkey groups (Table 3). This suggests that the disproportionally large volume of frontal lobe tracts is accompanied by a reduced volume fraction of some nonfrontal tracts, such as the anterior commissure. The absolute volume of this tract was significantly different between species (*F*_(2,12.91)_ = 89.85, *p* < 0.001) and was larger in humans than in the two monkey species (Table 3).

**Figure 4.**
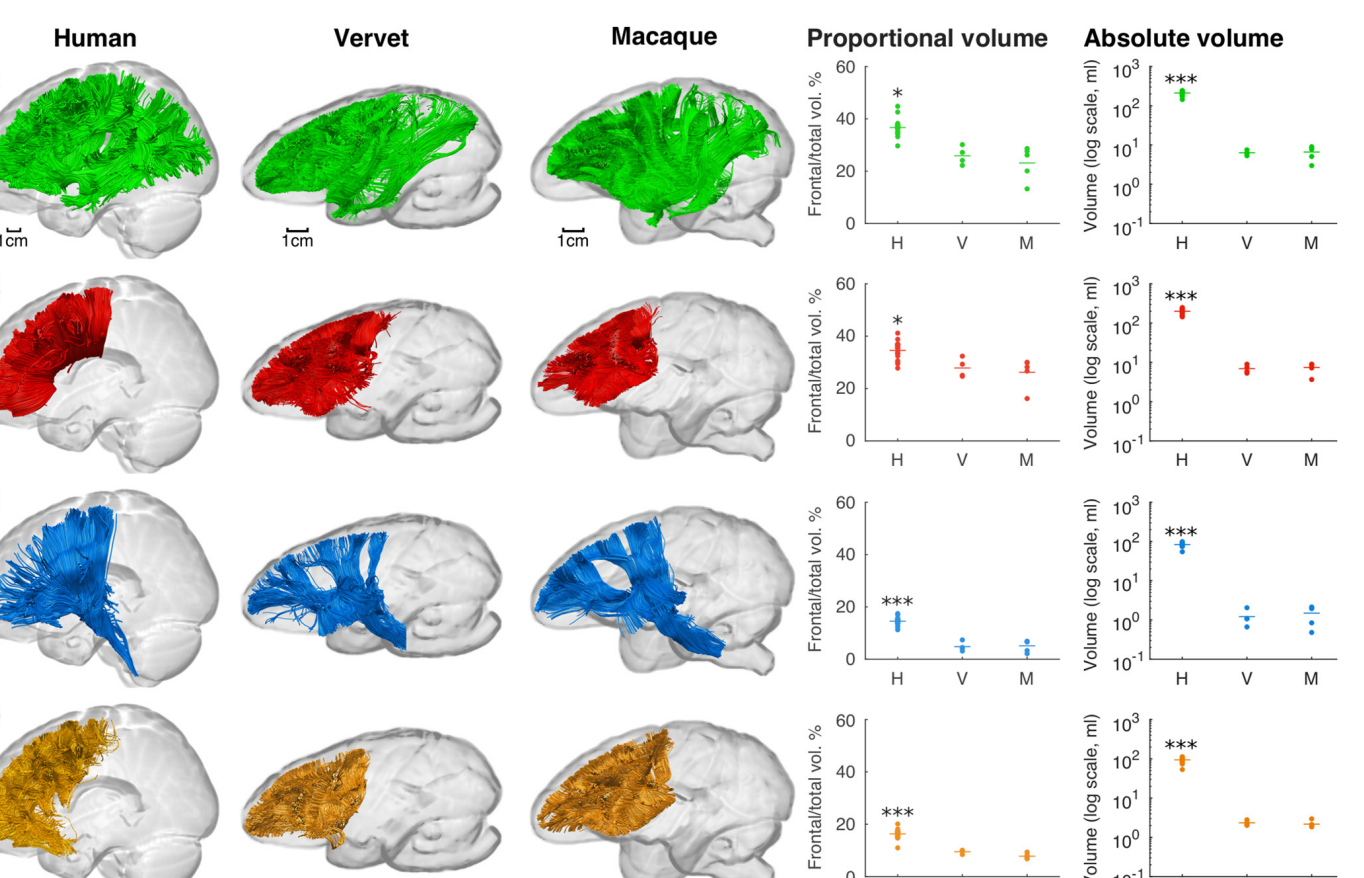
The main frontal tract groups compared among humans, vervets, and macaques. ***A***–***D***, Images show tractography reconstructions of the frontal association (green; ***A***), commissural (red; ***B***), projection (blue; ***C***), and intralobar frontal (orange; ***D***) networks in single representative brains. Graphs show both proportional volume and absolute volume of each tract group, where data points represent individual brains (H, *n* = 20; V, *n* = 4; M, *n* = 5) and species mean values are indicated by horizontal lines. *p < 0.05 and ***p < 0.001 when comparing humans with either vervets or macaques. For full statistical results, see Results and Table 4.

To understand whether the larger volume proportion of frontal white matter in humans compared with monkeys was attributable to a specific tract group or a general trend across all frontal lobe connections, volume measurements of the association, commissural and projection tract groups were obtained separately and compared across species (Fig. 4; Table 4). Statistically significant differences among the three groups were observed in the proportional frontal volume of the association (Welch's *F*_(2,5.54)_ = 22.06, *p* = 0.002), commissural (Welch's *F*_(2,5.67)_ = 42.56, *p* < 0.001), and projection (Welch's *F*_(2,5.65)_ = 71.14, *p* < 0.001) tract groups. *Post hoc* analysis shows that the frontal association tracts, which made up 36.69 ± 3.13% of the total white matter connection volume in humans, had a greater volume proportion compared with both vervets (25.92 ± 3.48%; *p* = 0.010) and macaques (23.15 ± 6.46%; *p* = 0.018). For the frontal commissural tracts, the volume fraction in humans (34.58 ± 3.30%) was higher than in vervets (27.85 ± 3.67%; *p* = 0.002) and macaques (26.19 ± 5.76%; *p* = 0.014). The projection tracts occupied 14.52 ± 1.44% of the total white matter volume in humans and only 4.80 ± 1.82% in vervets (*p* = 0.001) and 5.14 ± 2.25% in macaques (*p* = 0.001). In these three tract groups, no significant differences were found between the two monkey species. In addition, differences in proportional volume of the short intralobar association connections were detected (Welch's *F*_(2,9.52)_ = 113.33, *p* < 0.001), with humans showing higher values (16.33 ± 1.77%) compared with vervets (9.50 ± 0.73%; *p* < 0.001) and macaques (7.79 ± 1.04%; *p* < 0.001). Again, no differences were found between the two monkey species. These results suggest that differences between humans and monkeys in the volume of the frontal lobe pathways are attributable to a global change in both interlobar (i.e., association, commissural, and projections) and intralobar frontal connectivity. Absolute volumes of the above tract groups were also analyzed, revealing significantly larger volumes in humans and no significant differences between monkey species (association tracts: *F*_(2,10.95)_ = 535.787, *p* < 0.001; commissural tracts: *F*_(2,13.54)_ = 338.48, *p* < 0.001; projection tracts: *F*_(2,13.51)_ = 667.20, *p* < 0.001; intrafrontal tracts: *F*_(2,13.61)_ = 376.22, *p* < 0.001; Fig. 4; Table 4).

**Table 4.** Proportional and absolute frontal tract group volume measurements between species

Tract group	Human (mean ± SD)	Vervet (mean ± SD)	Macaque (mean ± SD)	*Post hoc* comparisons (*p* values)		
				Human versus vervet	Human versus macaque	Vervet versus macaque
Association						
Proportion (%)	36.69 ± 3.13	25.92 ± 3.48	23.15 ± 6.46	0.010	0.018	0.706
Absolute (ml)	211.92 ± 27.19	6.36 ± 0.92	6.64 ± 2.52	<0.001	<0.001	0.972
Commissural						
Proportion	34.58 ± 3.30	27.85 ± 3.67	26.19 ± 5.76	0.002	0.014	0.989
Absolute	200.42 ± 32.31	6.93 ± 1.67	7.42 ± 2.22	<0.001	<0.001	0.924
Projection						
Proportion	14.52 ± 1.44	4.80 ± 1.82	5.14 ± 2.25	0.001	0.001	0.937
Absolute	83.60 ± 9.78	1.22 ± 0.59	1.50 ± 0.78	<0.001	<0.001	0.818
Intrafrontal						
Proportion	16.33 ± 1.77	9.50 ± 0.73	7.79 ± 1.04	<0.001	<0.001	0.055
Absolute	94.53 ± 14.68	2.34 ± 0.34	2.17 ± 0.45	<0.001	<0.001	0.806

Association, commissural, projection, and intrafrontal tract group volumes in humans (*n* = 20), vervets (*n* = 4), and macaques (*n* = 5). Descriptive statistics and Games-Howell *post hoc* comparisons between species are given for proportional (normalized by total volume for each measure) and absolute volumes. See Results for Welch's ANOVA statistics.

**Figure 5.**
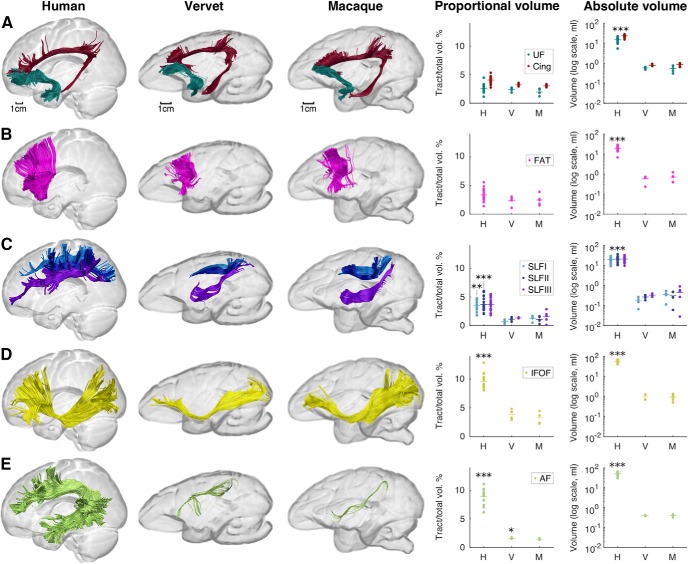
Comparison of the major frontal association tracts between humans, vervets, and macaques. Images show tractography reconstructions from individual brains, and graphs show proportional and absolute tract volume measures. Data points represent individual brains (H, *n* = 20; V, *n* = 4; M, *n* = 5). Species means are indicated by horizontal lines. ***A***–***E***, The tracts shown are the cingulum (burgundy color) and UF (dark green), which represent the major frontolimbic association tracts (***A***); FAT (pink; ***B***); frontoparietal connections of the superior longitudinal fasciculus (SLF I, light blue; SLF II, dark blue; SLF III, purple; ***C***); IFOF (yellow; ***D***); AF, long segment (light green; ***E***). *p < 0.05, **p < 0.01, and ***p < 0.001 when comparing humans with either vervets or macaques. For full statistical results, see Results and Table 5.

We then investigated differences between species in the main long association tracts, which included the cingulum, uncinate fasciculus, frontal aslant tract, superior longitudinal fasciculus, inferior fronto-occipital fasciculus, and the long segment of the arcuate fasciculus, using tractography dissections (Fig. 5; Table 5). There were no statistically significant differences between species in the cingulum, with volume fractions of 4.06 ± 0.62% in humans, 3.21 ± 0.29% in vervets, and 3.04 ± 0.23 in macaques (*F*_(2,5.55)_ = 3.00, *p* = 0.131); the uncinate fasciculus, with 2.56 ± 0.69% in humans, 2.38 ± 0.39% in vervets, and 1.97 ± 0.53% in macaques (*F*_(2,6.51)_ = 0.731, *p* = 0.517); or the frontal aslant tract, with 3.37 ± 1.00% in humans, 2.35 ± 0.86% in vervets, and 2.47 ± 0.92% in macaques (*F*_(2,6.68)_ = 3.01, *p* = 0.117). Significant differences in proportional volume were observed for all three branches of the superior longitudinal fasciculus. Branches I, II, and III occupied 3.46 ± 0.93%, 3.66 ± 1.17%, and 3.65 ± 1.08% of the total hemispheric white matter volume, respectively, in humans; 0.71 ± 0.36%, 1.12 ± 0.36%, and 1.33 ± 0.06%, respectively, in vervets; and 1.22 ± 0.44%, 1.06 ± 0.55%, and 1.54 ± 1.02%, respectively, in macaques (branch I: Welch's *F*_(2,9.71)_ = 54.13, *p* < 0.001; branch II: Welch's *F*_(2,10.20)_ = 40.12, *p* < 0.001; branch III: Welch's *F*_(2,9.04)_ = 27.78, *p* < 0.001). The inferior fronto-occipital fasciculus had volume proportions of 9.59 ± 1.22% in humans, 3.80 ± 0.89% in vervets, and 3.25 ± 0.94% in macaques (Welch's *F*_(2,7.30)_ = 101.22, *p* < 0.001), and most strikingly, the arcuate fasciculus had a proportional volume of 8.96 ± 1.38% in humans compared with 1.58 ± 0.11% in vervets and 1.45 ± 0.13% in macaques (Welch's *F*_(2,7.15)_ = 381.25, *p* < 0.001; Table 5). The absolute volumes of all the above tracts were significantly different (*p* < 0.001) between species (cingulum, *F*_(2,12.97)_ = 426.31; uncinate, *F*_(2,11.50)_ = 113.89; frontal aslant tract, *F*_(2,13.44)_ = 122.03; superior longitudinal fasciculus branch I, *F*_(2,12.80)_ = 110.79; branch II, *F*_(2,11.15)_ = 108.714; branch III, *F*_(2,9.28)_ = 98.28; inferior fronto-occipital fasciculus, *F*_(2,13.39)_ = 369.15; arcuate fasciculus, *F*_(2,12.83)_ = 214.42). The *post hoc* analysis shows that humans have significantly greater volume in all tracts than monkeys, and there are no significant differences between vervets and macaques (Table 5).

**Table 5.** Proportional and absolute volume measurements of frontal association tracts between species

Tract	Human (mean ± SD)	Vervet (mean ± SD)	Macaque (mean ± SD)	*Post hoc* comparisons (*p* values)		
				Human versus vervet	Human versus macaque	Vervet versus macaque
Cingulum						
Proportion (%)	4.06 ± 0.62	3.21 ± 0.29	3.04 ± 0.23			
Absolute (ml)	23.28 ± 3.35	0.79 ± 0.10	0.85 ± 0.16	<0.001	<0.001	0.760
UF						
Proportion	2.56 ± 0.69	2.38 ± 0.39	1.97 ± 0.53			
Absolute	14.86 ± 4.11	0.58 ± 0.08	0.56 ± 0.20	<0.001	<0.001	0.971
FAT						
Proportion	3.37 ± 1.00	2.35 ± 0.86	2.47 ± 0.92			
Absolute	19.26 ± 5.19	0.59 ± 0.24	0.71 ± 0.34	<0.001	<0.001	0.812
SLF I						
Proportion	3.46 ± 0.93	0.71 ± 0.36	1.22 ± 0.44	<0.001	0.001	0.225
Absolute	20.00 ± 5.85	0.17 ± 0.09	0.35 ± 0.15	<0.001	<0.001	0.142
SLF II						
Proportion	3.66 ± 1.17	1.12 ± 0.36	1.06 ± 0.55	<0.001	<0.001	0.986
Absolute	20.96 ± 6.09	0.27 ± 0.07	0.31 ± 0.18	<0.001	<0.001	0.902
SLF III						
Proportion	3.65 ± 1.08	1.33 ± 0.06	1.54 ± 1.02	<0.001	0.060	0.816
Absolute	21.07 ± 6.40	0.33 ± 0.05	0.46 ± 0.33	<0.001	<0.001	0.695
IFOF						
Proportion	9.59 ± 1.22	3.80 ± 0.89	3.25 ± 0.94	<0.001	<0.001	0.656
Absolute	55.34 ± 8.73	0.95 ± 0.30	0.92 ± 0.33	<0.001	<0.001	0.989
AF						
Proportion	8.96 ± 1.38	1.58 ± 0.11	1.45 ± 0.13	<0.001	<0.001	0.044
Absolute	52.07 ± 10.88	0.39 ± 0.03	0.40 ± 0.05	<0.001	<0.001	0.832

Individual frontal association tracts (cingulum; UF; FAT; SLF I, II, and III; IFOF; and AF) in humans (*n* = 20), vervets (*n* = 4), and macaques (*n* = 5). Descriptive statistics and Games-Howell *post hoc* comparisons between species are given for proportional (normalized by total volume for each measure) and absolute volumes in cases where signficant species-driven differences were observed with Welch's ANOVA. See results for ANOVA statistics.

**Figure 6.**
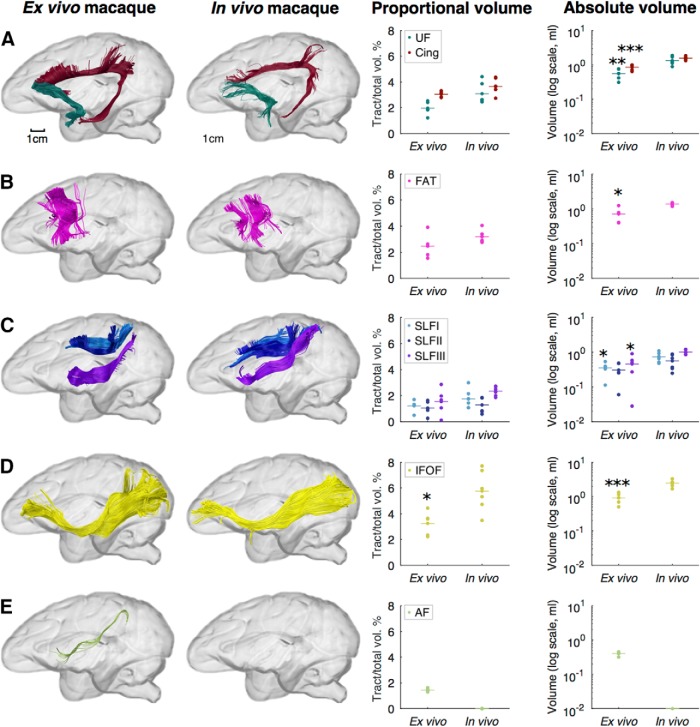
Comparison of *ex vivo* and *in vivo* macaque tractography data. ***A***–***E***, Images show tractography reconstructions of the cingulum (burgundy) and UF (dark green; ***A***), FAT (pink; ***B***), superior longitudinal fasciculus (SLF I, light blue; SLF II, dark blue; SLF III, purple; ***C***), IFOF (yellow; ***D***), and AF, long segment (light green; ***E***). Data points in the graphs show proportional and absolute tract volumes for individual brains and species mean values are indicated with horizontal lines. There were no significant differences in proportional tract volume between groups, except for the inferior fronto-occipital fasciculus (Welch's *F*_(1,6.08)_ = 8.34, *p = 0.027). *p < 0.05, **p < 0.01, and ***p < 0.001 when comparing ex vivo and in vivo macaques with Welch's ANOVA. The AF could not be reconstructed in the *in vivo* datasets. For full statistical results, see Table 6.

**Figure 7.**
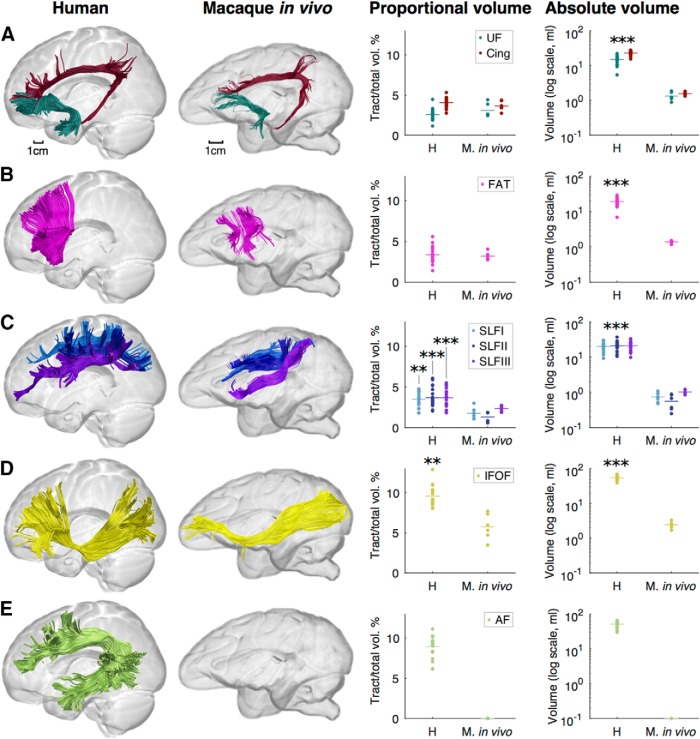
Comparison of human and macaque *in vivo* tractography data. ***A***–***E***, Images show tractography reconstructions of the cingulum (burgundy) and UF (dark green; ***A***), FAT (pink; ***B***), superior longitudinal fasciculus (SLF I, light blue; SLF II, dark blue; SLF III, purple; ***C***), IFOF (yellow; ***D***), and AF, long segment (light green; ***E***). Graphs show proportional and absolute tract volumes for individual brains measured from the *in vivo* dataset for both humans and monkeys. **p < 0.01 and ***p < 0.001 when comparing humans with either vervets or macaques. Statistics were not calculated for the AF because it was not possible to reconstruct this tract in the macaque *in vivo* datasets. For full statistical results, see Table 6.

Finally, we evaluated *in vivo* and *ex vivo* differences in our tractography volume measurements of the above tracts in macaques (Fig. 6; Table 6). We found no significant differences in volume proportions between *in vivo* and *ex vivo* macaques for the majority of tracts, including the cingulum, where the volume fraction in *in vivo* monkeys was 3.65 ± 0.61%; uncinate fasciculus, 3.09 ± 0.83%; frontal aslant tract, 3.20 ± 0.48%; and superior longitudinal fasciculus, where the volume proportion was 1.75 ± 0.74%, 1.29 ± 0.61%, and 2.35 ± 0.36% for branches I, II, and III, respectively. However, a significant difference was observed for the inferior fronto-occipital fasciculus proportional volume, which was 5.76 ± 1.60% in the *in vivo* macaque data compared with 3.25 ± 0.94% in the *ex vivo* data (Welch's *F*_(1,6.08)_ = 8.34, *p* = 0.027). The arcuate fasciculus was not included in this statistical comparison because it was not possible to reconstruct this tract in the in vivo macaque datasets, possibly due to insufficient spatial resolution. The absolute volumes were significantly different between *in vivo* and *ex* vivo macaques in all tracts analyzed except the superior longitudinal fasciculus III. To investigate interspecies differences within the same modality, we also compared human and macaque *in vivo* data (Fig. 7). Significant species differences were found in the three branches of the SLF, the IFOF and arcuate fasciculus, showing the same if not greater differences in tract volume proportions as seen in the human versus *ex vivo* monkey comparisons above. The absolute tract volumes were also significantly different between humans and *in vivo* monkeys for all tracts. Statistical comparisons are detailed in Table 6.

**Table 6.** Proportional and absolute *in vivo* volume measurements of frontal association tracts in macaques compared with *ex vivo* macaques and *in vivo* humans

Tract	*In vivo* macaques	Comparison with *ex vivo* macaques			Comparison with *in vivo* humans		
		Welch's *F*	df within groups	*p*	Welch's *F*	df within groups	*P*
Cingulum							
Proportion (%)	3.65 ± 0.61	0.84	7.18	0.388	1.45	5.76	0.276
Absolute (ml)	1.57 ± 0.18	46.44	8.91	<0.001	830.60	19.37	<0.001
UF							
Proportion	3.09 ± 0.83	4.18	7.96	0.075	2.07	5.26	0.207
Absolute	1.34 ± 0.37	19.35	7.87	0.002	210.49	20.01	<0.001
FAT							
Proportion	3.20 ± 0.48	2.83	6.21	0.142	0.10	13.01	0.757
Absolute	1.37 ± 0.12	16.86	4.83	0.010	237.00	19.07	<0.001
SLF I							
Proportion	1.75 ± 0.74	1.14	7.68	0.318	15.71	6.92	0.006
Absolute	0.74 ± 0.24	10.54	8.60	0.011	215.52	19.21	<0.001
SLF II							
Proportion	1.29 ± 0.61	0.01	7.95	0.921	43.33	12.49	<0.001
Absolute	0.56 ± 0.28	3.36	8.55	0.102	222.56	19.26	<0.001
SLF III							
Proportion	2.35 ± 0.36	1.34	4.42	0.306	19.15	22.37	<0.001
Absolute	1.01 ± 0.16	11.92	5.50	0.016	196.12	19.08	<0.001
IFOF							
Proportion	5.76 ± 1.60	8.34	6.08	0.027	19.73	4.98	0.007
Absolute	2.46 ± 0.55	32.61	8.37	<0.001	724.84	19.49	<0.001

Individual frontal association tracts (cingulum, UF; FAT; SLF I, II, and III; and IFOF) in *in vivo* macaques (*n* = 6). The arcuate fasciculus could not be reconstructed in *in vivo* macaques. Descriptive statistics and *F*, within-groups degrees of freedom (df), and *p* values are given. In all cases, the between-groups df = 1. Welch's ANOVA was used to compare *in vivo* with *ex vivo* macaques, and *in vivo* with humans. Results are presented for proportional (normalized by total volume for each measure) and absolute volumes.

## Discussion

Two main findings emerged from our study. First, the larger proportional volume of frontal connections in humans compared with monkeys is driven by association, commissural, projection, and intrafrontal networks, suggesting greater communication within and between the frontal and other lobes in our species. Second, within the association tracts, species differences were driven by tracts important for motor planning, top-down visual and auditory processing, auditory memory, and language. No significant differences were observed in tracts involved mainly in emotional processing and social behaviour, such as the cingulum and uncinate fasciculus.

One novel dimension of our study was to consider the full extent of connections between the frontal and other lobes. Conventional voxel-based and tissue-sectioning techniques only measure white matter within the frontal lobes, whereas tractography analyzes networks extending throughout the brain. In our study, tractography revealed larger proportional volumes of local and extended frontal networks in humans compared with monkeys. This result is in line with voxel-based analyses in the present study and in the literature (Schoenemann et al., 2005; Smaers et al., 2011) and emphasizes the role of the frontal lobes in functions that rely on distributed networks (Smaers et al., 2017; Donahue et al., 2018). Evidence suggests that this result is driven by prefrontal rather than premotor and motor frontal connections (Smaers et al., 2017). Given the larger proportion of frontal white matter in humans than monkeys, we demonstrated the converse to be true for some nonfrontal tracts, as seen with the anterior commissure. This finding aligns with previous studies demonstrating a significantly smaller anterior commissure cross-sectional area in humans than monkeys (Foxman et al., 1986; Rilling and Insel, 1999).

In addition, we demonstrated that the greater proportional volume of human frontal connections was true of association, projection, and commissural tract groups. This is consistent with previous reports suggesting that cortico-ponto-cerebellar connections (Ramnani et al., 2006; Smaers and Vanier, 2019) and the anterior corpus callosum (Catani and Thiebaut de Schotten, 2012) receive proportionally larger contributions from prefrontal areas in humans compared with monkeys. Among the association pathways, greater frontal connectivity was documented in humans for both intralobar and interlobar tracts, suggesting more cross talk not only within frontal areas, but also between frontal and nonfrontal areas.

Furthermore, our analysis of individual long association tracts revealed unique features of human white matter connectivity, with the arcuate fasciculus showing the most striking species differences. Nonhuman primates share a small subcomponent of the arcuate fasciculus with humans, projecting to the posterior superior temporal gyrus, consistent with previous macaque axonal tracing (Petrides and Pandya, 2002; Schmahmann and Pandya, 2006) and diffusion imaging studies (Croxson et al., 2005; Rilling et al., 2008). This subcomponent is thought to be involved in acoustic spatiotemporal processing and stimulus identification (Aboitiz and García, 2009). However, in humans, the long segment of the arcuate fasciculus projects more anteriorly to the superior temporal gyrus and extends to the middle and inferior temporal gyri (Catani et al., 2005; Thiebaut de Schotten et al., 2012), which are proportionally larger in humans. The arcuate fasciculus links perisylvian regions involved with auditory memory (Rauschecker and Scott, 2009; Schulze et al., 2012), word learning (López-Barroso et al., 2013), and syntax (Wilson et al., 2011).

Another tract with significant differences between species was the inferior fronto-occipital fasciculus. Although the functions of this tract remain largely unknown (Forkel et al., 2014), its greater proportional volume in humans may facilitate direct frontal access to visual inputs and top-down control of early visual processing for functions such as face and object perception (Pins and ffytche, 2003; Bar et al., 2006) and reading (Shaywitz et al., 2002). It is important to note that the existence of this tract in monkeys is debated, and most visual associative areas in the human occipital lobe are located in temporal and parietal lobes of the monkey brain. Tractography (Mars et al., 2016; Feng et al., 2017) and blunt dissection studies (Decramer et al., 2018; Sarubbo et al., 2019) show connections between frontal and occipital lobes in monkeys, matching the trajectory of the inferior fronto-occipital fasciculus in humans (Curran, 1909). However, neither of these methods is able to distinguish monosynaptic from polysynaptic pathways, leaving open the question of whether these pathways are direct connections or composed of segments with lateral terminations in the temporal cortex. The question arises because many axonal tracing studies, which have been able to identify monosynaptic pathways, have failed to reveal the inferior fronto-occipital fasciculus (Schmahmann and Pandya, 2006; Petrides, 2013). Other macaque axonal tracing studies have revealed connections between frontal and occipital cortices (Barbas and Mesulam, 1981; Gerbella et al., 2010; Markov et al., 2014); however, their methods are not sensitive to axonal trajectories, and they do not report whether these axons follow the course expected for the inferior fronto-occipital fasciculus. Further investigation is required to resolve this issue, which in our view is primarily due to a posterior shift in the human brain of many visual areas located in the parietal and temporal lobes of the monkey brain.

Differences in the superior longitudinal fasciculus were also significant. These frontoparietal tracts are involved in motor cognition (Duffy and Burchfiel, 1971; Leiguarda and Marsden, 2000; Parlatini et al., 2017) and visuospatial attention (Corbetta et al., 2002; Picard and Strick, 2003; Buschman and Miller, 2007; Goldenberg and Spatt, 2009; Thiebaut de Schotten et al., 2011; Parlatini et al., 2017). Their damage manifests with visuospatial neglect (Beis et al., 2004; Thiebaut de Schotten et al., 2014) and impaired reaching and grasping in humans and monkeys (Leiguarda and Marsden, 2000), suggesting common functions across species. Indeed, the superior longitudinal fasciculus provides parietal input to the superior premotor cortex (Petrides and Pandya, 1984), part of an interconnected frontal network for hand and digit movement (Dum and Strick, 2002, 2005; Howells et al., 2018; Hopkins and Phillips, 2017). Beyond manual dexterity, interspecies differences in this tract may be related to functions greatly developed in humans, such as tool making (Hecht et al., 2015) and writing (Duncan, 2010; Purcell et al., 2011; Planton et al., 2013; Genovesio et al., 2014).

The lack of species differences in the uncinate fasciculus and cingulum indicates a shared anatomical substrate for these frontolimbic tracts dedicated to aspects of memory (Gaffan and Wilson, 2008), decision-making (Rushworth and Behrens, 2008), and social and emotional behavior (Rolls, 2015). Similarly, a lack of differences in the frontal aslant tract, a recently described pathway between the inferior frontal gyrus and superior medial frontal cortex (Lawes et al., 2008; Catani et al., 2012b), may indicate a common substrate for vocalization or orofacial movements (Petrides et al., 2005).

To verify that interspecies differences in our results were not driven by *in vivo–ex vivo* differences, we compared both modalities within macaques and investigated species differences with *in vivo* data. The *in vivo–ex vivo* comparison showed overall agreement in proportional volume, whereas absolute volume was greater *in vivo*, possibly due to *ex vivo* tissue shrinkage or greater partial volume effects in the lower-resolution *in vivo* datasets. Our comparison of *in vivo* human and macaque data showed similar interspecies differences to the main results. We therefore favored using *ex vivo* monkey datasets in our analysis over lower-resolution *in vivo* data to maximize our ability to resolve small white matter bundles in the monkey brain.

While tractography is the only method currently able to reconstruct white matter pathways *in vivo* (Dell'Acqua and Catani, 2012; Jbabdi et al., 2015), its limitations are widely acknowledged (Jones, 2010; Dell'Acqua and Catani, 2012; Dell'Acqua and Tournier, 2019). We used deterministic rather than probabilistic tractography to avoid tract length and direction biases (Jones, 2010; Liptrot et al., 2014; Donahue et al., 2016), whole-brain seeding to prevent initialization point bias, and spherical deconvolution to estimate multiple fiber directions per voxel (Dell'Acqua et al., 2010; Jones, 2010; Catani et al., 2012a). To minimize false positives (Maier-Hein et al., 2017), tractography was inspected by an expert anatomist (M.C.), and streamlines with anatomically implausible trajectories were manually removed.

In this study, we focused on the frontal lobe; however, other areas of association cortex play equally significant roles in human high-order functions. Temporal and parietal regions are also shown to be disproportionally larger in humans than monkeys (Van Essen and Dierker, 2007), although the prefrontal cortex appears to show the greatest difference (Smaers et al., 2017). Accordingly, in our results, the frontal tracts with the greatest species differences in volume proportion were those connecting with temporal, parietal, and occipital association areas. In the future, the networks of other lobes should be studied more fully to understand differences between human and nonhuman primates (Catani et al., 2017).

In conclusion, diffusion tractography revealed a greater proportional volume of frontal white matter networks in humans compared with monkeys, with significant differences in association, commissural, projection, and intrafrontal networks. Striking interspecies differences were found for the arcuate, superior longitudinal and inferior fronto-occipital fasciculi. Other frontal association tracts and one nonfrontal limbic tract, the anterior commissure, occupied similar or smaller volume proportions in humans compared with monkeys. Although we were unable to make inferences about evolution directly, these results support the hypothesis of rearrangement of whole-brain connectivity during human evolution. This pattern of long-range frontal connectivity in humans may have resulted from reduced reliance on certain limbic functions, increased feedforward relay of sensory inputs, and direct top-down modulation of early perceptual processing necessary for the development of higher cognitive functions.
